# A machine learning and remote sensing approach for accurate forest sub-compartment level vegetation cover change monitoring

**DOI:** 10.3389/fpls.2026.1741992

**Published:** 2026-01-26

**Authors:** Wenjie Zhang, Yingze Tian, Xiaohui Su, Danzi Wu

**Affiliations:** 1 School of Information Science & Technology, Beijing Forestry University, Beijing, China; 2 Forestry Information Research Institute, Beijing Forestry University, Beijing, China; 3 Sichuan Forestry and Grassland Survey and Planning Institute, Chengdu, China; 4 School of Landscape Architecture, Beijing Forestry University, Beijing, China

**Keywords:** change detection, forest sub-compartment (FSC), machine learning, remote sensing image, texture feature

## Abstract

**Introduction:**

Accurate detection of vegetation cover type changes in forest sub-compartments (FSCs) is essential for supporting informed forest management decisions. Although various forest change detection algorithms have been developed, fine-scaledetection at the FSC level has received limited attention.

**Methods:**

This study addresses this gap by developing an FSC-scale vegetation cover type change detection method that couples spectral and texture information from Sentinel-2 multispectral imagery with forest management planning and design investigation (FMPI) data. Original spectral bands, vegetation indices, and texture features were extracted and used to construct a classification model based on a particle swarm optimization–back propagation neural network (PSO-BPNN). To evaluate performance, the proposed PSO-BPNN method was compared with random forest (RF), support vector machine (SVM), and conventional back-propagation neural network (BPNN) models.

**Results:**

Results indicate that PSO-BPNN consistently outperformed the other algorithms in change detection at the FSC scale. Specifically, the method achieved an overall accuracy of 91% for change identification, with a Kappa coefficient of 0.86. In the validation dataset, it successfully detected approximately 80% of the changed FSCs.

**Discussion:**

The proposed approach offers a robust and accurate solution for fine-scale forest change monitoring, it enhances the scientific basis for sustainable forest resource management.

## 1 Introduction

A forest sub-compartment (FSC) is the basic operational unit for forest management planning and design investigation (FMPI). It represents a forest plot with relatively homogeneous internal characteristics and clear boundaries that distinguishing it from neighboring units. In China’s forest resource inventory system, FSCs are typically delineated based on basic conditions such as land type, dominant tree species, and forest type, with their boundaries usually defined by identifiable topographic features. The area of an FSC generally ranges from 5 to 20 hectares, depending on management intensity, and the minimum area is determined by whether it can be represented on a basic map. The basic information on forest area, resources, and biomass collected in FMPI is organized at the FSC level. FSC surveys are therefore a critical component for forest management units in formulating management plans and for forestry authorities in conducting resource assessments (Wu et al., 2012). For a long time, monitoring FSC changes has relied primarily on reports submitted by operating units. However, irregular disturbances—such as illegal land-use changes, fires, pests, and diseases—as well as underreporting of management activities, have made accurate and timely FSC change monitoring a persistent challenge. Traditionally, FSC change information has been obtained through manual interpretation of remote sensing imagery, field inspections, or periodic forest resource surveys conducted at intervals of up to ten years. These approaches are labor-intensive, costly, and often subject to temporal delays and uncertainty. Consequently, obtaining FSC change information in a timely, accurate, and efficient manner, and updating FSC datasets accordingly, remains a key technical challenge faced by forest management authorities (Zhao et al., 2002).

The advancement of remote sensing technology has created new opportunities for forest monitoring. However, in current forestry practice, forest management staff still primarily rely on visual interpretation, overlaying remote sensing imagery from two periods to make a preliminary judgment on FSC changes. For areas where changes cannot be clearly determined or remain disputed, field investigations are required. These field verifications not only confirm the occurrence of changes but also update essential information such as land type, causes, and other relevant attributes. Integrating the verified field data ultimately produces a complete database of FSC change information. Given the current forestry workflow and operational practices, a critical challenge remains: how to effectively use remote sensing imagery to automatically and accurately extract FSC change information, thereby reducing misjudgment and inefficiency caused by the subjectivity of manual interpretation (Panigrahy et al, 2010). The demand for up-to-date and reliable information on vegetation cover type changes has fueled rapid progress in change detection methods and algorithms based on remote sensing data. The proliferation of Earth observation datasets, advances in remote sensing technologies, the rise of machine learning algorithms, and the expansion of high-performance computing capabilities have collectively accelerated research in change detection and driven continuous innovation in detection methodologies (Hussain et al, 2013; Cheng et al, 2024).

Over the past decades, a variety of forest change detection methods have been proposed. The direct classification method directly classifies multi-temporal images and is one of the most common approaches for detecting forest changes using multi-temporal satellite data and harmonic analysis. Leveraging time-series remote sensing data, several well-established algorithms have been widely applied, including Breaks For Additive Season and Trend (BFAST) (Masiliunas et al, 2021), Landsat-based detection of Trends in Disturbance and Recovery (LandTrendr) (Kennedy et al, 2010), Continuous Change Detection and Classification (CCDC) (Zhu and Woodcock, 2014), Vegetation Change Tracker (VCT) algorithm (Huang et al, 2010). However, algorithms relying on long time series imply that a large amount of data needs to be downloaded, organized, stored and processed, with high computational cost, complex data processing, and monitoring accuracy is greatly affected by the length of the time series, and the stability of the variables (Gao et al, 2021). With the rise of artificial intelligence, deep learning methods have emerged as powerful tools for remote sensing change detection. For instance, Zhao et al. (2022) proposed U-Net to generate a map of deforestation hotspots by comparing post-harvested patches with the surrounding intact forest using Sentinel-1 data. Zhang et al. (2021) fused Landsat and Sentinel-2, and generated a scaling method based on deep learning to generate forest disturbance maps with a lot of spatial details for tracking small-scale disturbances in tropical forests. Deep learning demonstrates substantial potential in remote sensing change detection; however, it requires large volumes of high-quality training data, which are often difficult to obtain promptly within a specific region of interest. Furthermore, when applied to high-resolution remote sensing imagery, these methods are accompanied by significantly increased computation time (Zhang et al., 2020).

Post-classification change detection methods, which compare classification results from different time periods, remain among the most widely used techniques (Li, 2003; Tewkesbury et al, 2015). For example, the Pan-European High-Resolution Change Layers—such as Impervious Surface Density or Tree Cover Density from the Copernicus Land Monitoring Service—are produced from single-date products collected at three-year intervals. Forest change surveys draw on multiple data sources, including satellite imagery, aerial photography, airborne laser scanning, or combinations thereof. Medium-resolution imagery such as Landsat and Sentinel-2, owing to their free availability, global coverage, and frequent updates, are among the most widely adopted (Bullock et al, 2020; Chen et al, 2021; Novo-Fernandez et al, 2018). At the scale of FSC, typically ranging from 5 to 20 hectares, changes are often subtle and small in spatial extent, making them challenging to detect using medium-resolution satellite imagery. This scale-specific challenge requires the development of more sophisticated detection methods that integrate time series data, multi-source feature extraction (such as spectral and texture information), and advanced machine learning algorithms to improve the accuracy and efficiency of forest cover change detection.

Recent studies indicate a clear shift from pixel-based approaches toward FSC or object-based frameworks, primarily because key forest inventory variables, such as area, forest resources, and aboveground biomass, are defined and managed at the irregular FSC scale. Forest stands within an FSC generally exhibit internal consistency and similarity, resulting in more homogeneous pixels and richer remote sensing information when aggregated at the FSC level. Compared with pixel-based methods, FSC-based approaches exploit within-stand homogeneity to enhance the reliability of vegetation information extraction, making them better aligned with operational forest inventory and management practices (Liu et al., 2023). Beyond forest change detection (Zhou et al., 2025), recent FSC-scale studies have extended to forest aboveground biomass estimation (Lv et al., 2023), forest health and disease monitoring (Liu et al., 2023; Zhang et al., 2025), and vegetation type assessment (Zhang et al., 2025), all of which provide critical indicators of vegetation cover dynamics. These advances highlight a growing trend toward integrated FSC-scale monitoring frameworks, with future prospects focused on automated, data-rich, and operationally applicable support for forest management.

This study focuses on addressing the key scientific challenge currently facing change detection at the FSC scale: how to build an efficient change detection process applicable to multi-temporal data to meet the dual needs of forest management for timeliness and accuracy. In this study, we address these challenges by proposing a FSC-scale vegetation cover type change detection method that couples spectral and texture features extracted from Sentinel-2 imagery with FMPI data. The specific objectives of this study are as follows: (1) Feature Selection for FSC Change Detection: Spectral, vegetation index, and texture features from Sentinel-2 satellite imagery were systematically extracted and evaluated to identify the most informative variables to enhance classification and change detection performance at the FSC scale. (2) Development of an optimized change detection model: A particle swarm optimization–back propagation neural network (PSO-BPNN) model was constructed for FSC vegetation cover type change detection and benchmarked against Random Forest (RF), Support Vector Machine (SVM), and Backpropagation Neural Network (BPNN) methods. (3) Regional-scale FSC vegetation mapping and trend analysis: Detailed regional-scale FSC vegetation cover type maps were constructed and the spatiotemporal patterns of vegetation dynamics were assessed. This study provides a comprehensive and effective framework for accurate FSC-scale vegetation cover change detection by integrating feature selection, advanced modeling, and regional-scale analysis, thereby supporting improved forest management and monitoring.

## 2 Materials and methods

### 2.1 Study area

**Figure 1:**
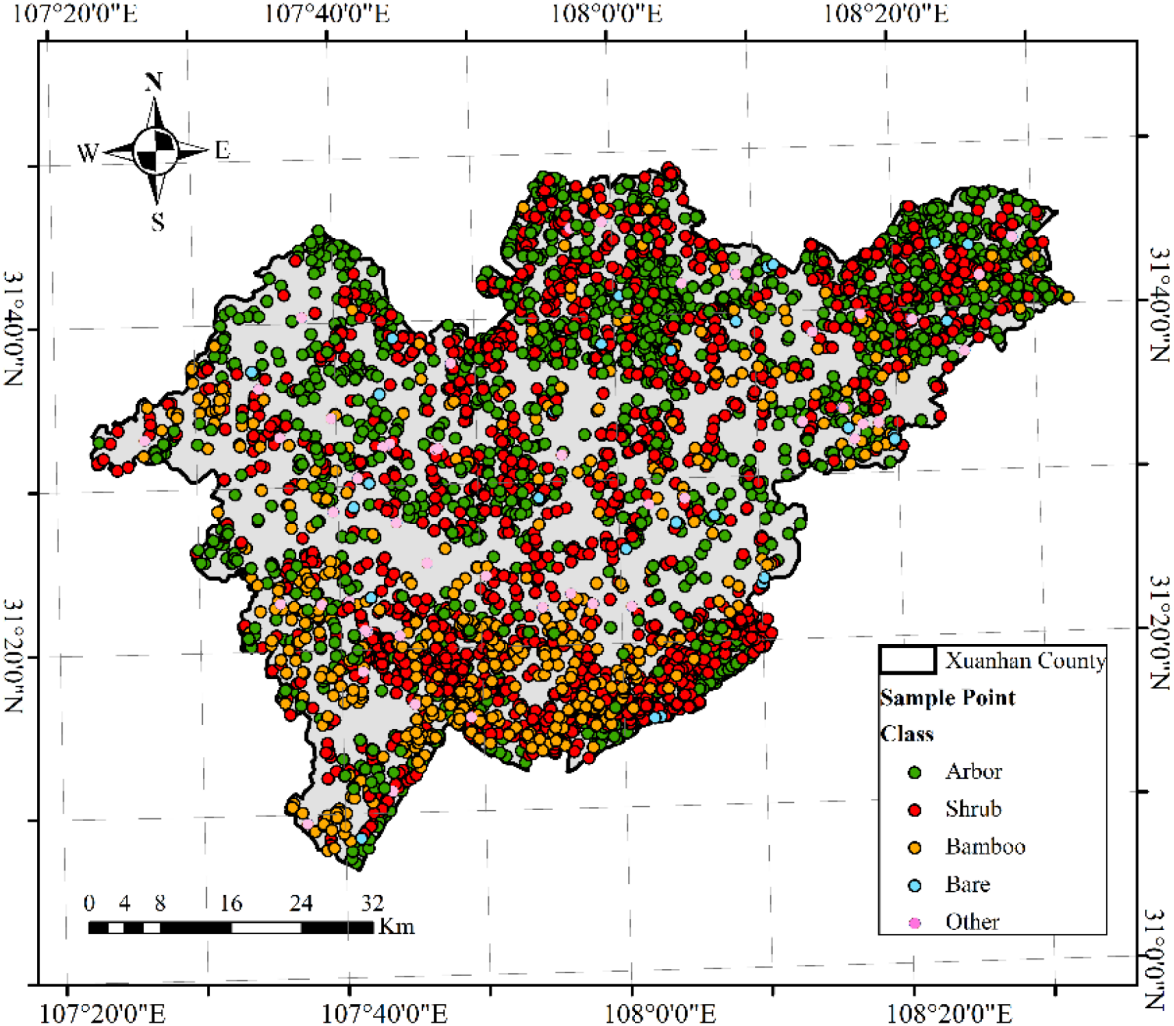
Location of the study area and distribution of FSCs. The sampling points represent the different vegetation cover types of the FSC samples in the training sample.

The study area is located in Xuanhan County, Dazhou City, Sichuan Province, at the southern foot of the Daba Mountains in the Sichuan Basin, between longitudes 107°20′ and 108°20′ E and latitudes 31°00′ and31°50′ N. The region has a mid-subtropical humid monsoon climate, with an average annual temperature of approximately 16.8°C, an average annual precipitation of 1230 mm, and a frost-free period of approximately 296 days. The region’s topography is complex, with altitudes ranging from an average of 780 meters to a maximum of 2458 meters. The vegetation is categorized into five typical cover types: trees, shrubs, bamboo forests, bare land, and other woodlands (**Figure 1**). Dominant tree species include Chinese fir and Masson pine, collectively covering over 70% of the area. Other notable species include cypress *(Cupressus funebris Endl.*), oak (*Quercus acutissima*), and pine *(Pinus kesiya Royle ex Gordon*).

### 2.2 Dataset and preprocessing

#### 2.2.1 Field data

The ground survey data used in this study consist of two independent datasets. The first dataset is the FSC data of the Sichuan Province FMPI in 2019~2021, which are utilized for FSC boundary extraction, vegetation type determination, and accuracy evaluation. The second dataset is the “One Map of Forest Land” data of Sichuan Province for 2019~2021, which integrates remote sensing interpretation, official land-use approval records, afforestation project archives, and field verification of changed FSCs, and is primarily used to provide documented causes of FSC changes.

The study area contains a total of 340588 FSCs, with individual FSC area ranging from 100 m^2^ to 224,289 m^2^, and the dataset contains attribute fields such as area, vegetation cover type, vegetation cover type change in 2019 to 2021, dominant tree species, geomorphology, elevation, and so on. The vegetation cover types in the study area are classified into five major categories. The change reason attribute is derived from the “One Map of Forest Land” dataset and reflect field-verified or officially documented causes of change, including afforestation projects, tree removal associated with construction land use, natural vegetation succession, and disturbance events.

To develop a robust change detection model, improve classification stability, and reduce noise introduced by very small patches, only FSC samples with an area larger than 3,000 m^2^ were selected for training. Furthermore, to ensure representativeness and training efficiency, the proportion of training samples for each vegetation cover type in the study area was adjusted based on its abundance while avoiding excessive class imbalance. The detailed composition of the FSC samples is presented in **Table 1**.

**Table 1:** Statistical overview of FSC land types and vegetation cover types in the study area.

Type	Definition	Entire study area		Training dataset	
		Quantity	Proportion	Quantity	Proportion
Arbor	Composition of arbor tree species, canopy density ≥ 0.2	319107	93.69	1803	43.87
Shrub	Composition of shrub species, coverage ≥ 40%	17132	5.03	1645	40.02
Bamboo	Bamboo species with a diameter of ≥ 2cm, canopy density ≥ 0.2	2726	0.80	574	13.97
Bare	Vegetation cover ≤ 10%, and there is no obvious seasonal vegetation recovery	1194	0.35	30	0.73
Other	Not belonging to the other three types of land, this study specifically refers to grassland and bare land	429	0.13	58	1.41

#### 2.2.2 Remote sensing data

Google Earth Engine (GEE) is a powerful cloud computing platform launched by Google in 2010. It excels in data acquisition, analysis and processing. Compared with traditional remote sensing image processing software such as ENVI 5.3 and ArcGIS 10.8, GEE has obvious advantages. The platform provides numerous open source codes, including publicly available datasets and free storage space. It integrates datasets from multiple satellite platforms, including Landsat, MODIS, Sentinel, SRTM, DMSP and NPP VIIRS, totaling more than 200 datasets. These features make GEE an important tool for remote sensing image processing. The Sentinel-2 data used in this paper is from the GEE Open Access Data Center. Sentinel-2 provides multispectral imagery across 13 spectral bands, from visible to shortwave infrared, with spatial resolutions of 10 m, 20 m, and 60 m, depending on the selected spectral band. This study used RGB bands with a spatial resolution of 10 m.

Sentinel-2 images for the growing season (spring, summer, and autumn) of each year from 2019 to 2021 were acquired from the GEE platform and processed for multi-temporal synthesis to obtain composite images with the lowest cloud cover and good radiometric consistency during that period. All images used for synthesis were limited to cloud cover below 10%. Areas with cloud cover above 10% were defined as areas with large cloud cover and were removed in subsequent processing. The Sentinel-2 L1C data product used has completed radiometric and geometric correction, where radiometric correction includes terrain correction and atmospheric correction based on a digital elevation model. Due to the large scope of the study area, some Sentinel-2 images still have large areas of cloud cover. In the subsequent training and prediction process, these areas with thicker clouds will be masked out to reduce their impact on classification and change detection. The main reason for choosing growing season imagery is that vegetation is in its most vigorous growth phase during this period, and the spectral reflectance characteristics and differences between different vegetation types are most significant, which helps improve the discriminative capabilities of classification and change detection. Furthermore, the high vegetation cover during the growing season effectively reduces interference from soil, litter, and other non-vegetation background, thereby improving the overall accuracy and stability of the model.

#### 2.2.3 Dataset preparation

Due to the irregular boundaries of FSCs, the spatial location of sample extraction can substantially influence vegetation cover change detection results. To improve classification accuracy and reduce the impact of mixed pixels and boundary-related spectral ambiguity between adjacent FSCs, a centroid-based central image extraction strategy was adopted in this study.

First, the ground survey shapefile layer was used to spatially mask the Sentinel-2 imagery. Then, the Geospatial Data Abstraction Library (GDAL) *gdalwarp* tool was applied to clip the imagery at the FSC level. Considering the 10 m × 10 m spatial resolution of Sentinel-2 data and the fixed patch-based model input, FSCs with an area larger than 3,000 m² were used for model training to ensure sufficient interior pixels for representative sampling and to reduce boundary-related interference during learning. For each FSC, the geometric centroid was calculated, and a fixed-size image patch of 5 × 5 pixels (corresponding to 50 m × 50 m at the Sentinel-2 spatial resolution) was extracted by centering the window on this centroid location. This patch size was selected to provide a consistent spatial context for model input while minimizing boundary effects and mixed-pixel contamination. For FSCs exceeding 3,000 m², the centroid-based patch is highly likely to be effectively enclosed within the FSC interior, thereby implicitly reducing the influence of boundary effects.

By adopting a fixed-size, centroid-based extraction strategy, spatial consistency among samples was maintained, which is beneficial for stable model training and reliable performance evaluation. FSCs smaller than 3,000 m² were not discarded; instead, they were excluded from training but retained in the testing stage and treated as out-of-distribution samples to evaluate model generalization performance for FSCs approaching the spatial-resolution limit of Sentinel-2 imagery. The resulting central image dataset was subsequently divided into training (80%) and testing (20%) subsets for model development and accuracy assessment.

### 2.3 Methods

**Figure 2:**
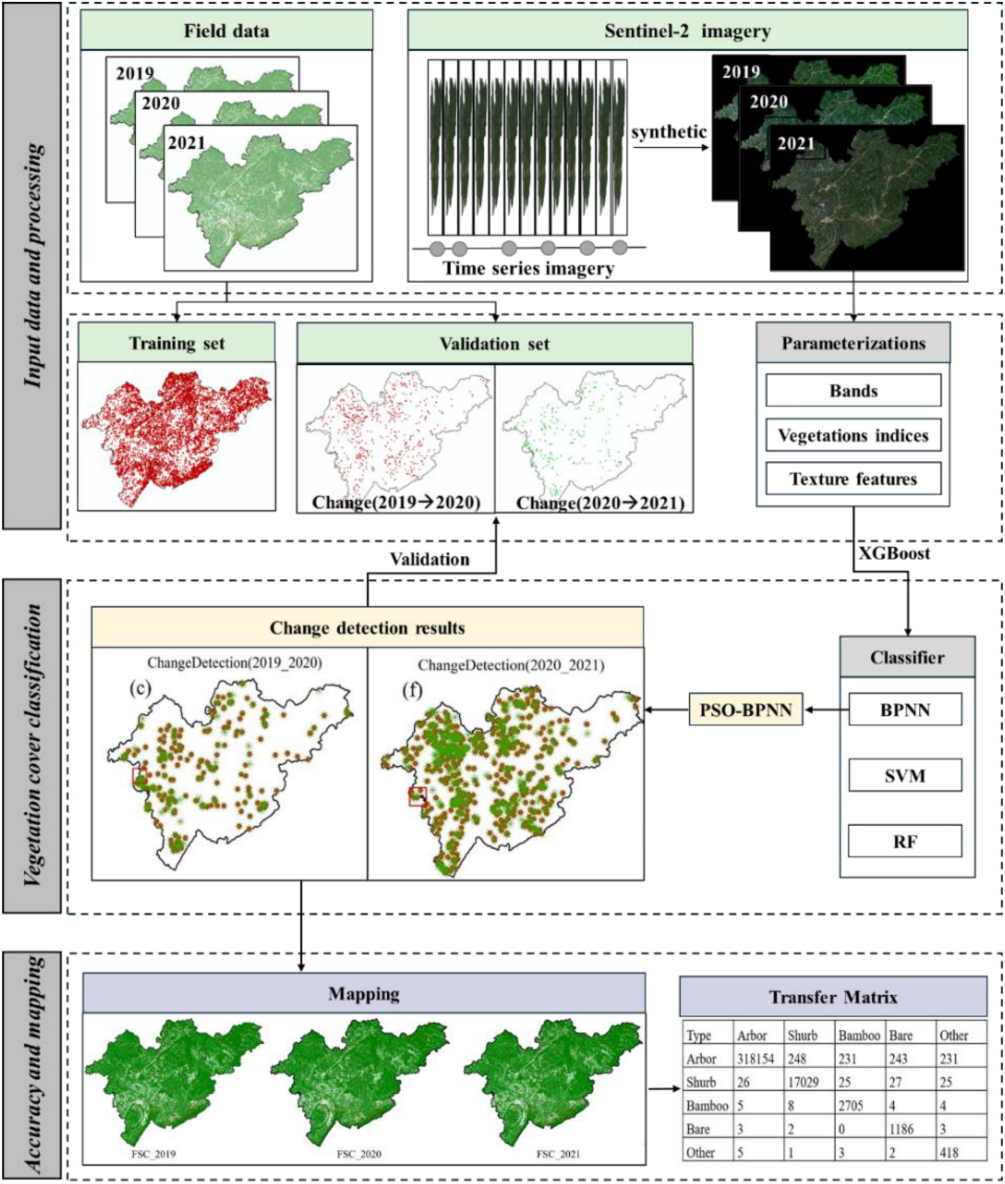
Flowchart of FSC vegetation cover type change detection based on Sentinel-2 images.

The overall technical workflow of this study (**Figure 2**) consists of five main stages. First, data collection and preprocessing were performed by acquiring Sentinel-2 multi-temporal imagery and ground survey data from 2019 to 2021, and the training and test datasets were divided. Second, multi-feature construction and selection were performed. Original spectral bands, vegetation indices, and gray level co-occurrence matrix (GLCM) texture features were extracted from the preprocessed imagery, and feature screening was performed. Third, classification models were developed and compared using three classifiers. The particle swarm optimization (PSO) algorithm was used to optimize the weight initialization and parameter search of the BPNN to improve model convergence and classification accuracy. Fourth, post-classification change detection and accuracy assessment were performed. Multi-temporal classification results were compared to identify changes in forest cover types. Accuracy was verified using field survey data. Finally, change mapping and results analysis were performed, and a vegetation type transition matrix was calculated to quantify transitions between types.

#### 2.3.1 Features extraction and Selection

This study comprehensively utilizes raw spectra, vegetation indices, and texture features to improve the accuracy and robustness of ground object classification. Specifically, three types of feature information were extracted based on Sentinel-2 multispectral remote sensing images. The details are as follows:

(1) Raw spectral bands.

Sentinel-2 provides a series of multispectral bands across the visible to shortwave infrared range. In this study, four key spectral bands were selected for feature extraction, including B2 (Blue), B3 (Green), B4 (Red), and B8 (Near-Infrared, NIR), all with 10-meter spatial resolution. These bands were chosen due to their sensitivity to vegetation phenology and structural characteristics.

(2)Vegetation indices for change detection.

Based on the literature review, 10 common spectral vegetation indices (VIs), i.e., normalized vegetation index (NDVI) and enhanced vegetation index (EVI), etc., were derived by using the GEE platform based on the sensitivity of optical features to the vegetation cover type. The complete list of formulas is shown in **Table 2**.

**Table 2:** Vegetation indices for change detection.

Vegetation index	Code	Formulation	Reference
Normalized Difference Vegetation Index	NDVI	(B8−B4)/(B8+B4)	(Rouse et al, 1973)
Enhanced Vegetation Index	EVI	2.5×((B8−B4)/(B8+6×B4−7.5×B2+1))	(Huete et al, 2002)
Normalized Burn Ratio Index	NBRI	(B8−B12)/(B8+B12)	(Key and Benson, 2006)
Soil-Adjusted Vegetation Index	SAVI	1.5×((B8−B4)/(B8+B4+0.5))	(Huete, 1988)
Ratio Vegetation Index	RVI	B8/B4	(Jordan, 1969)
Difference Vegetation Index	DVI	B8−B4	(Richardson and Wiegand, 1977)
Normalized Difference Built-up Index	NDBI	(B11−B8)/(B11+B8)	(Zha et al., 2003)
Blue-Green-Red Vegetation Index	BGRVI	(B3−(B2+B4))/(B3+(B2+B4))	(Tucker, 1979)
Green Normalized Difference Vegetation Index	GNDVI	(B8−B3)/(B8+B3)	(Gitelson et al, 1996)
Normalized Difference Water Index	NDWI	(B3−B8)/(B3+B8)	(McFeeters, 1996)

(3) Texture Analysis of Images.

Texture describes the spatial patterns and relationships of image pixels and is important for image analysis and classification (Farwell et al., 2021). GLCM is a matrix function containing pixel distances and angles that detects the gray level distribution of neighboring pixels by considering the spatial location of the pixel in the image (Fu et al., 2021). In this study, we selected eight standard GLCM-based texture features as shown in **Table 3**.

**Table 3:** Texture metrics formulas.

Texture metrics	Code	Formulation *	Application
Mean	MEAN	$\sum_{\text{i},\text{j}}\text{i}\times \text{P}\left(\text{i},\text{j}\right)$	Weighted calculation of average processing window values based on the frequency of occurrence of pixel values combined with specific neighborhood pixel values (Held, 1998)
Variance	VAR	$\sum_{\text{i},\text{j}}\frac{{\left(\text{P}\left(\text{i},\text{j}\right)-\text{μ}\right)}^{2}}{\text{N}-1}$	Reflects the contours and gray scale changes of each homogeneous region of the image
Homogeneity	HOM	$\sum_{\text{i},\text{j}}\frac{\text{P}\left(\text{i},\text{j}\right)}{1+\left|\text{i}-\text{j}\right|}$	Measuring the level of homogeneity of pixel pairs (Humeau-Heurtier, 2019)
Contrast	CON	$\sum_{\text{i},\text{j}}{\left|\text{i}-\text{j}\right|}^{2}\text{P}\left(\text{i},\text{j}\right)$	Measures the amount of local texture change in an image (Christaki et al, 2022)
Dissimilarity	DIS	$\sum_{\text{i},\text{j}}\text{iP}\left(\text{i},\text{j}\right)\left|\text{i}-\text{j}\right|$	Reflecting image heterogeneity
Entropy	ENT	$-\sum_{\text{i},\text{j}}\text{P}\left(\text{i},\text{j}\right){\text{log}}_{2}\text{P}\left(\text{i},\text{j}\right)$	Evaluating GLCM disorder, reflecting the complexity of texture distribution (Zhang et al., 2017)
Energy	ENE	$\sum_{\text{i},\text{j}}\text{P}{\left(\text{i},\text{j}\right)}^{2}$	Measures the uniformity of the texture in the image
Correlation	COR	$\frac{\sum_{\text{i},\text{j}}\left(\text{i}-\bar{\text{x}}\right)\left(\text{j}-\bar{\text{y}}\right)\text{P}\left(\text{i},\text{j}\right)}{{\text{σ}}_{\text{x}}{\text{σ}}_{\text{y}}}$	Measure the linear relationship of gray levels between pixels

* i and j in the table are the number of rows and columns of the image, respectively, and P is the number of grayscale covariance matrices in the GLCM.

To select the most discriminative variables for classification tasks from a high-dimensional feature set, this study used the ensemble learning algorithm eXtreme Gradient Boosting (XGBoost) for feature selection. XGBoost constructs a series of weighted regression tree models, effectively evaluating the contribution of each feature to optimizing the loss function during model training. Considering the semantic differences among feature types, spectral features (including raw spectral bands and VIs) and texture features were treated as two independent feature groups during the ranking process. This separation avoids potential bias caused by differences in feature dimensionality or feature quantity between groups and allows each type of information to be evaluated within a comparable context. For each feature group, candidate variables were ranked according to their gain values, and the most representative features were identified based on their relative importance and cumulative contribution, rather than by applying a fixed numerical threshold. Although spectral bands and VIs derived from similar wavelengths may exhibit mutual correlation, tree-based ensemble models such as XGBoost are generally robust to multicollinearity. In this study, correlated features were evaluated jointly during the ranking process, and redundant variables tended to receive lower gain scores. Consequently, the selected spectral and texture features were subsequently fused and used as input for the classification model.

#### 2.3.2 Machine learning classifications

In this study, a machine learning-based approach was adopted to classify vegetation types using Sentinel-2 multispectral remote sensing imagery. Three commonly used classifiers— BPNN, SVM, and RF—were first employed and systematically compared. Based on classification accuracy and model stability, the optimal base classifier was further enhanced using PSO, resulting in an optimized hybrid model, PSO-BPNN.

The RF model was configured with 50 decision trees (n_estimators=50) and a maximum tree depth of 8 (max_depth=8). The random seed was fixed at 42 (random_state=42) to ensure reproducibility. The SVM classifier adopted the Radial Basis Function (RBF) kernel (kernel=‘rbf’). A grid search strategy was employed to tune the penalty parameter C (explored in the range 0.1 to 100; here selected as C = 100) and the kernel coefficient *y* (gamma=‘scale’, corresponding to an adaptive default). Probability estimation was enabled (probability=True). Feature scaling was performed prior to training to improve model convergence. The BPNN model was constructed as a three-layer feedforward neural network with one input layer, two hidden layers (each with 50 neurons), and one output layer. The input layer size matched the number of input features (input_size), and the output layer size corresponded to the number of vegetation classes (num_classes). The ReLU activation function was applied in the hidden layers, and the Adam optimizer was used for training. The model was trained for 300 epochs to ensure convergence. Hyperparameters such as the number of hidden neurons (range 16–128) and learning rate (0.001–0.1) were explored in preliminary experiments to determine optimal values.

**Figure 3:**
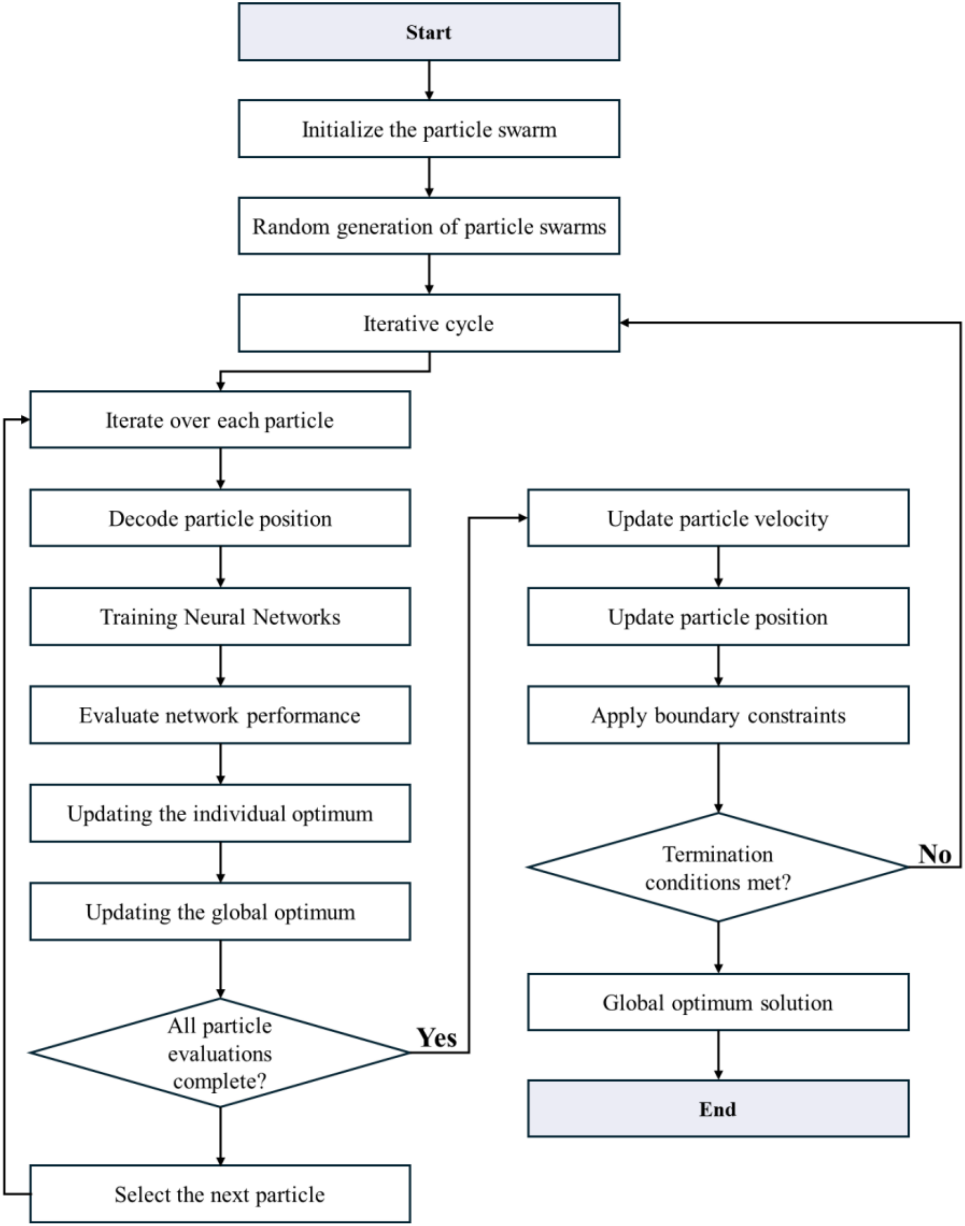
Workflow of the PSO-BPNN model integrating particle swarm optimization with backpropagation neural network training.

To address limitations of traditional BPNN including slow convergence and local minima entrapment, PSO was integrated to optimize the initial weights and biases. The PSO algorithm initialized a swarm of 50 particles and iteratively updated their positions over 100 generations, following commonly adopted configurations in PSO-based neural network studies in remote sensing (Tamiminia et al., 2017; Hamedianfar et al., 2022). Learning factors were set to c_1_ = c_2_ = 1.5, and the inertia weight w was linearly decreased from 0.9 to 0.4 during optimization. The optimal number of neurons in the two hidden layers, optimal_layer1 and optimal_layer2, were determined by PSO and subsequently used to define the final BPNN architecture. Given the moderate dimensionality of the input feature space, the selected PSO parameter settings were sufficient to achieve stable convergence behavior while maintaining computational efficiency. Repeated experimental runs indicated consistent optimization performance under these settings. This hybrid approach significantly improved convergence speed and model generalization. In addition, the workflow of the PSO-BPNN model was illustrated in a flowchart (see **Figure 3**), which clearly depicts the integration of particle swarm optimization with the backpropagation neural network training process.

#### 2.3.3 Accuracy assessment

To quantitatively evaluate the classification performance of the machine learning models, multiple commonly used metrics were adopted, including Overall Accuracy (OA), Precision, Recall, F1-score, and the Kappa coefficient. These metrics comprehensively reflect both the predictive accuracy and the model’s robustness across different vegetation types. These evaluation metrics were calculated based on the confusion matrix derived from the validation dataset. By comparing the performance of different classifiers using these indicators, the most reliable and generalizable model for vegetation classification was identified. The evaluation metrics were calculated using the formulas presented in Equations 1–5, as follows:

(1) $$\text{OA}=\frac{\text{TP}+\text{TN}}{\text{TP}+\text{TN}+\text{FP}+\text{FN}}$$

(2) $$\text{Precision}=\frac{\text{TP}}{\text{TP}+\text{FP}}$$

(3) $$\text{Recall}=\frac{\text{TP}}{\text{TP}+\text{FN}}$$

(4) $$\text{F}1-\text{score}=\frac{2\times \text{Precision}\times \text{Recall}}{\text{Precision}+\text{Recall}}$$

(5) $$\text{Kappa}=\frac{{\text{P}}_{\text{o}}-{\text{P}}_{\text{e}}}{1-{\text{P}}_{\text{e}}}$$

TP is true positive; TN is true negative; FP is false positive; FN is false negative; N is total number of samples; P_o_ is the observed overall agreement rate; P_e_ is the random agreement rate.

## 3 Results

### 3.1 Importance of independent variables

**Figure 4:**
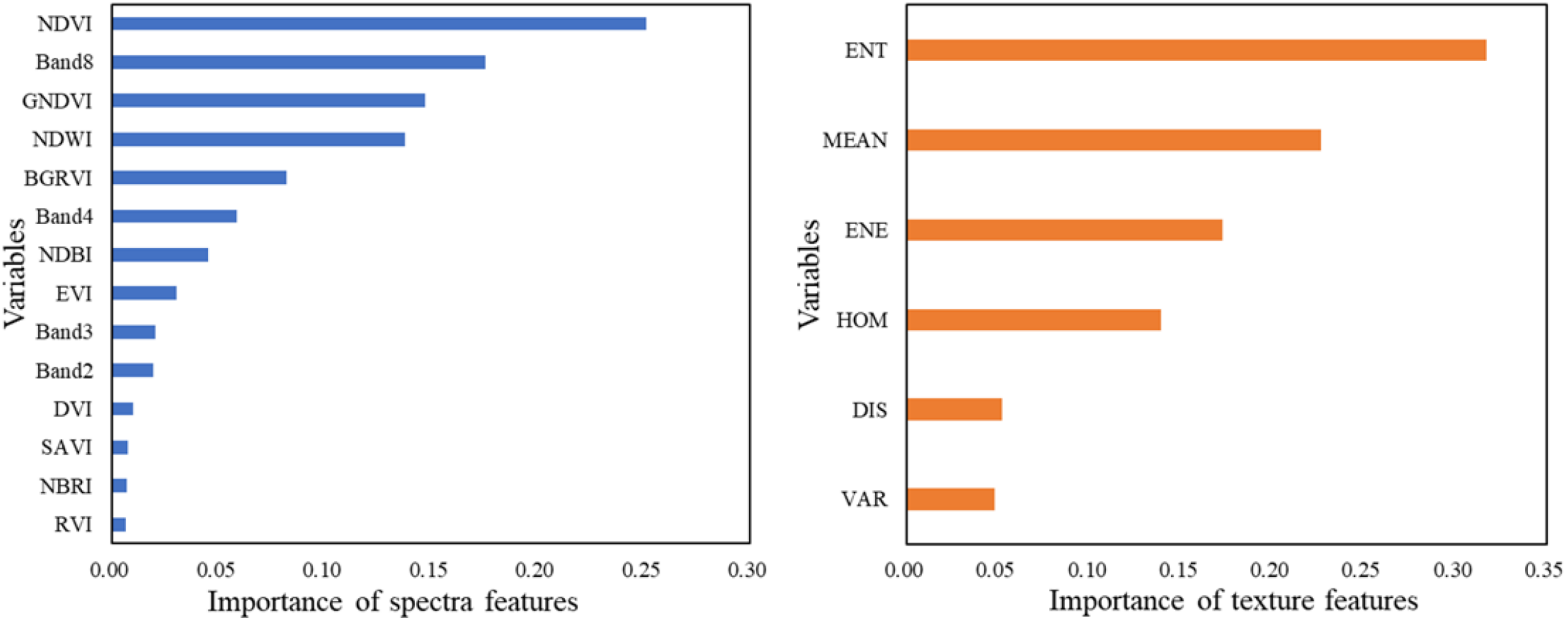
Evaluation of importance of features.

To identify the most discriminative features for the classification task, the XGBoost algorithm was employed to assess the importance of all candidate features. **Figure 4** presents the feature importance rankings derived from the XGBoost model, where each bar represents the relative contribution of a given feature to the training process, quantified by the gain—defined as the average information gain achieved by the feature when used to split nodes across all trees. The results indicate that among the original spectral bands, the near-infrared band B8, along with several vegetation indices such as NDVI, GNDVI, and NDWI, exhibit high importance scores, underscoring their strong discriminative power for differentiating land cover types and their critical role in remote sensing classification.

Furthermore, to evaluate the contribution of spatial structural information to classification performance, GLCM texture features were analyzed separately for their importance. The findings reveal that texture metrics including ENT, MEAN, and ENE are highly significant, collectively accounting for 85.6% of the cumulative importance. This high importance is attributed to their ability to effectively characterize the spatial heterogeneity and structural complexity of land surfaces, which often cannot be fully captured by spectral information alone. Specifically, entropy measures the randomness or disorder within the image texture, mean reflects the average intensity, and energy quantifies textural uniformity; together, they provide complementary information about surface patterns and variations. Consequently, ENT, MEAN, and ENE were selected as the primary texture features for subsequent classification modeling.

In this study, comparative experiments were conducted using three different input feature combinations: (I) Sentinel-2A spectral bands only (Band), (II) spectral bands combined with vegetation indices (Band + VIs), and (III) spectral bands, vegetation indices, and GLCM texture features (Band + VIs + Texture). The results (**Table 4**) show that the choice of input features has a substantial effect on classification performance.

**Table 4:** Effects of the combination of spectral band, vegetation index and GLCM texture features on the classification accuracy of RF, SVM, BPNN and PSO-BPNN.

Input features	RF		SVM		BPNN		PSO-BPNN	
	OA	Kappa	OA	Kappa	OA	Kappa	OA	Kappa
Band	0.70	0.60	0.72	0.61	0.72	0.61	0.76	0.65
Band + VIs	0.75	0.64	0.77	0.67	0.78	0.69	0.85	0.76
Band + VIs + Texture	0.79	0.71	0.81	0.72	0.85	0.76	0.91	0.86

When using only the bands, the OA ranged from 0.70 (RF) to 0.76 (PSO-BPNN), with Kappa coefficients between 0.60 and 0.65. Adding VIs produced consistent improvements for all classifiers, with OA increases of approximately 5–9% and Kappa coefficient increases of about 0.04–0.11. The PSO-BPNN model showed the largest improvement in this stage, with OA rising from 0.76 to 0.85 and Kappa from 0.65 to 0.76. Further incorporating GLCM texture features led to the highest classification accuracies across all models, with OA reaching 0.79–0.91 and Kappa values ranging from 0.71 to 0.86. In particular, PSO-BPNN achieved the best overall performance, with OA improving from 0.76 (Band) to 0.91 and Kappa from 0.65 to 0.86—representing an overall gain of 15% in OA and 0.21 in Kappa. These results highlight the strong adaptability of PSO-BPNN to multi-source feature fusion. Overall, the integration of spectral, vegetation index, and texture features significantly enhances classification accuracy and class separability, particularly in complex terrain and heterogeneous vegetation conditions. The inclusion of texture information captures spatial heterogeneity more effectively, thereby improving vegetation mapping precision and increasing the reliability of the final classification results.

### 3.2 Comparison of classification algorithm accuracy

**Figure 5:**
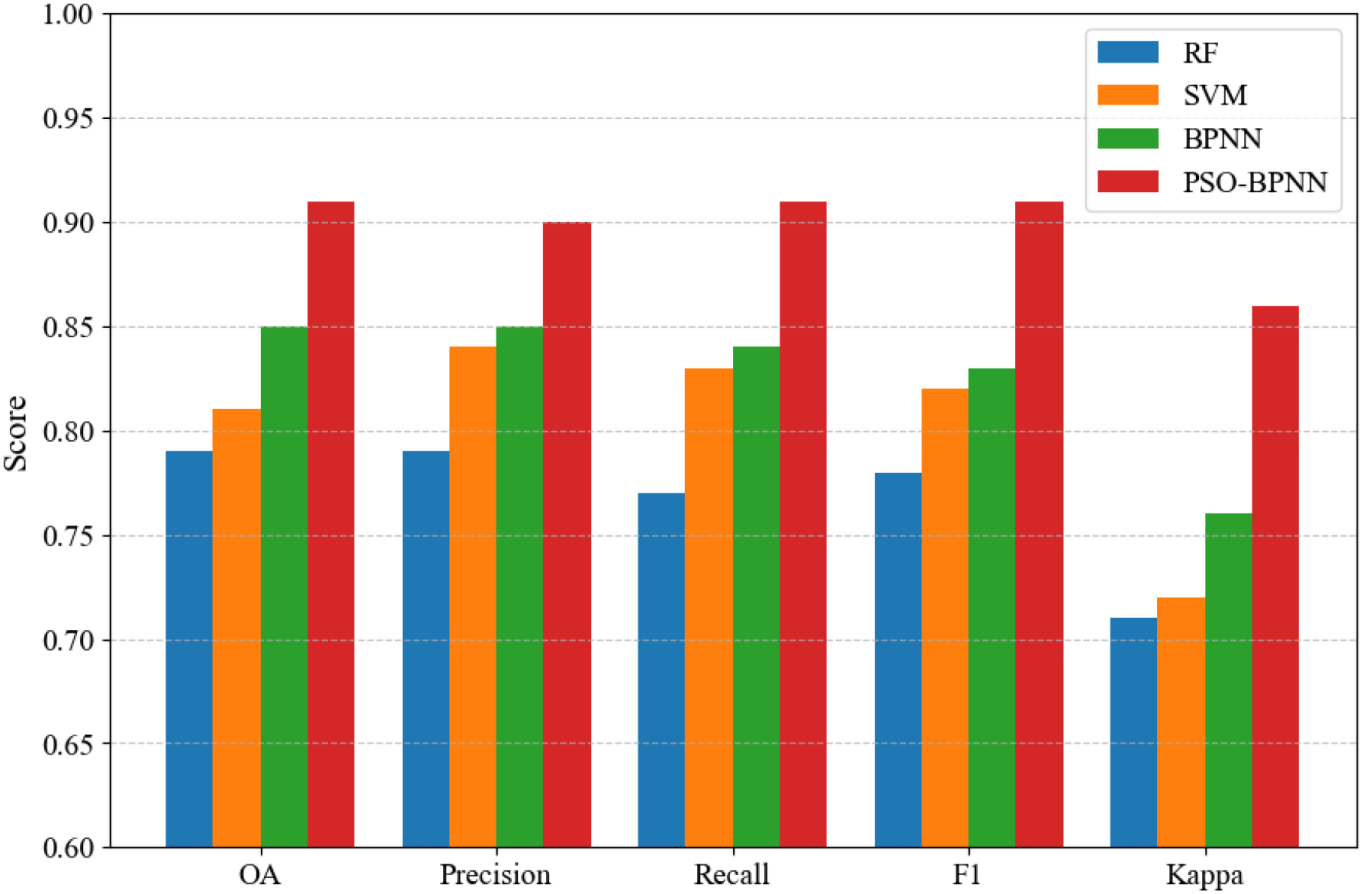
Comparative evaluation of RF, SVM, BPNN, and PSO-BPNN in classification accuracy metrics.

**Figure 5** illustrates the comparative performance of the four classification models evaluated in this study. The models were assessed on the validation set using OA, precision, recall, F1-score, and the Kappa coefficient. Among these models, the PSO-BPNN achieved the highest performance, with an OA of 0.91, notably exceeding those of BPNN (0.85), SVM (0.81), and RF (0.79). It also demonstrated high precision (0.90), recall (0.91), and F1-score (0.91), indicating strong recognition capability and a well-balanced ability to identify both positive and negative cases. Moreover, the Kappa coefficient reached 0.86, reflecting excellent classification consistency and robustness in multi-class classification tasks.

**Figure 6:**
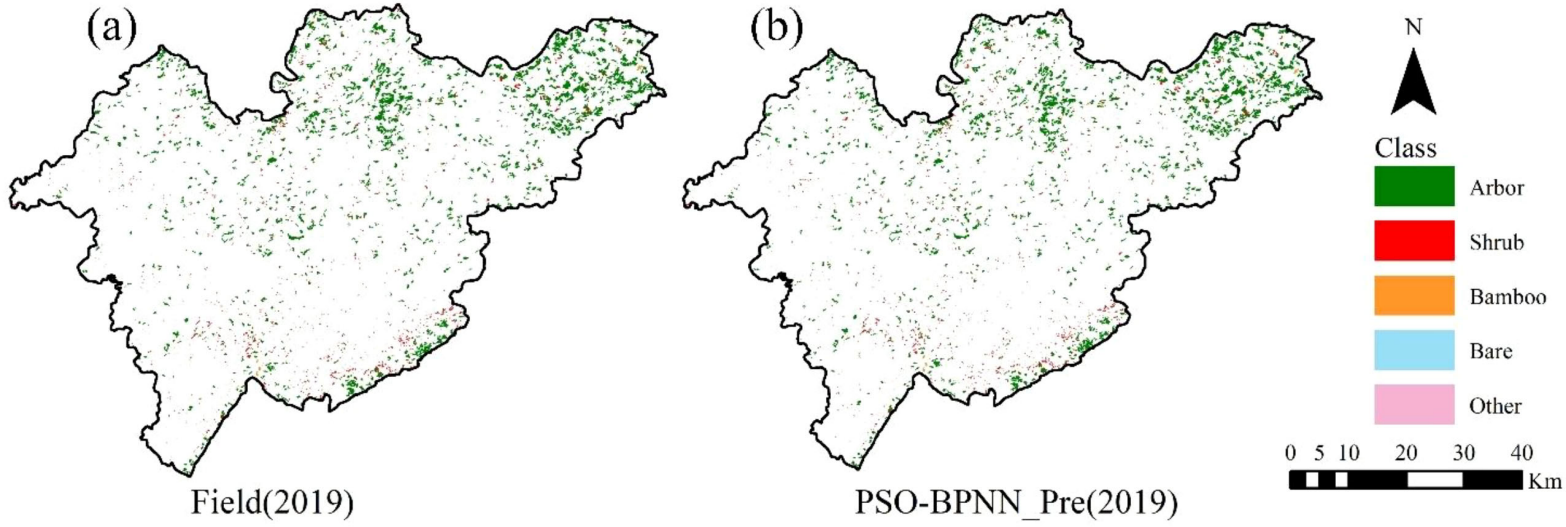
2019 field survey results and PSO-BPNN model classification results. **(a)** Spatial distribution of vegetation cover types based on the 2019 field survey; **(b)** spatial distribution of vegetation cover types predicted for 2019 using the PSO-BPNN model.

The application of Particle Swarm Optimization significantly improved the traditional BPNN’s performance, increasing OA from 0.85 to 0.91 and Kappa from 0.76 to 0.86. This demonstrates PSO’s effectiveness in optimizing network parameters and enhancing global convergence. In contrast, SVM and RF models exhibited lower OA (≤0.81) and Kappa coefficients (≤0.72), suggesting reduced robustness in handling class imbalance and samples with fuzzy boundaries. To further validate the model’s applicability, a spatial comparison was performed between the 2019 field survey results and the PSO-BPNN classification outputs (**Figure 6**). The spatial distribution patterns show high agreement, with the PSO-BPNN model accurately capturing fine-scale variations in vegetation cover types. These results confirm that PSO-BPNN possesses strong spatial generalization capability, making it a reliable and effective choice for vegetation classification tasks.

**Figure 7:**
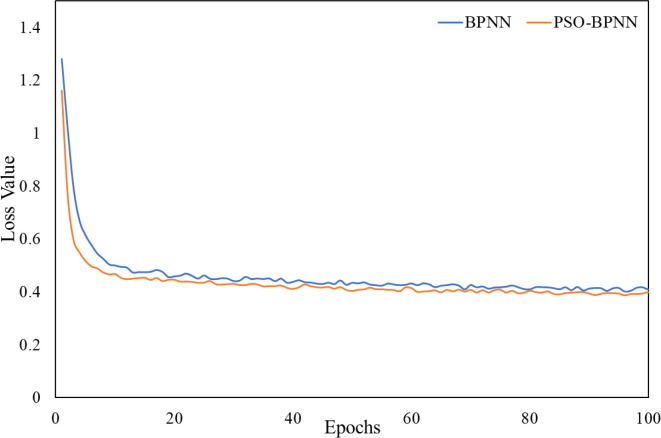
Training loss curves for the BPNN and PSO-BPNN.

Additionally, the loss function curves for BPNN and PSO-BPNN during training are presented in **Figure 7**. The PSO-BPNN model exhibited lower loss values from the early training stages and converged more rapidly with smaller fluctuations, ultimately stabilizing at a markedly lower loss level than the unoptimized BPNN. These results indicate that incorporating the particle swarm optimization algorithm improves weight initialization and parameter search efficiency, thereby enhancing training stability, accelerating convergence, and strengthening the model’s generalization capability.

### 3.3 Results of vegetation cover type change detection

**Figure 8:**
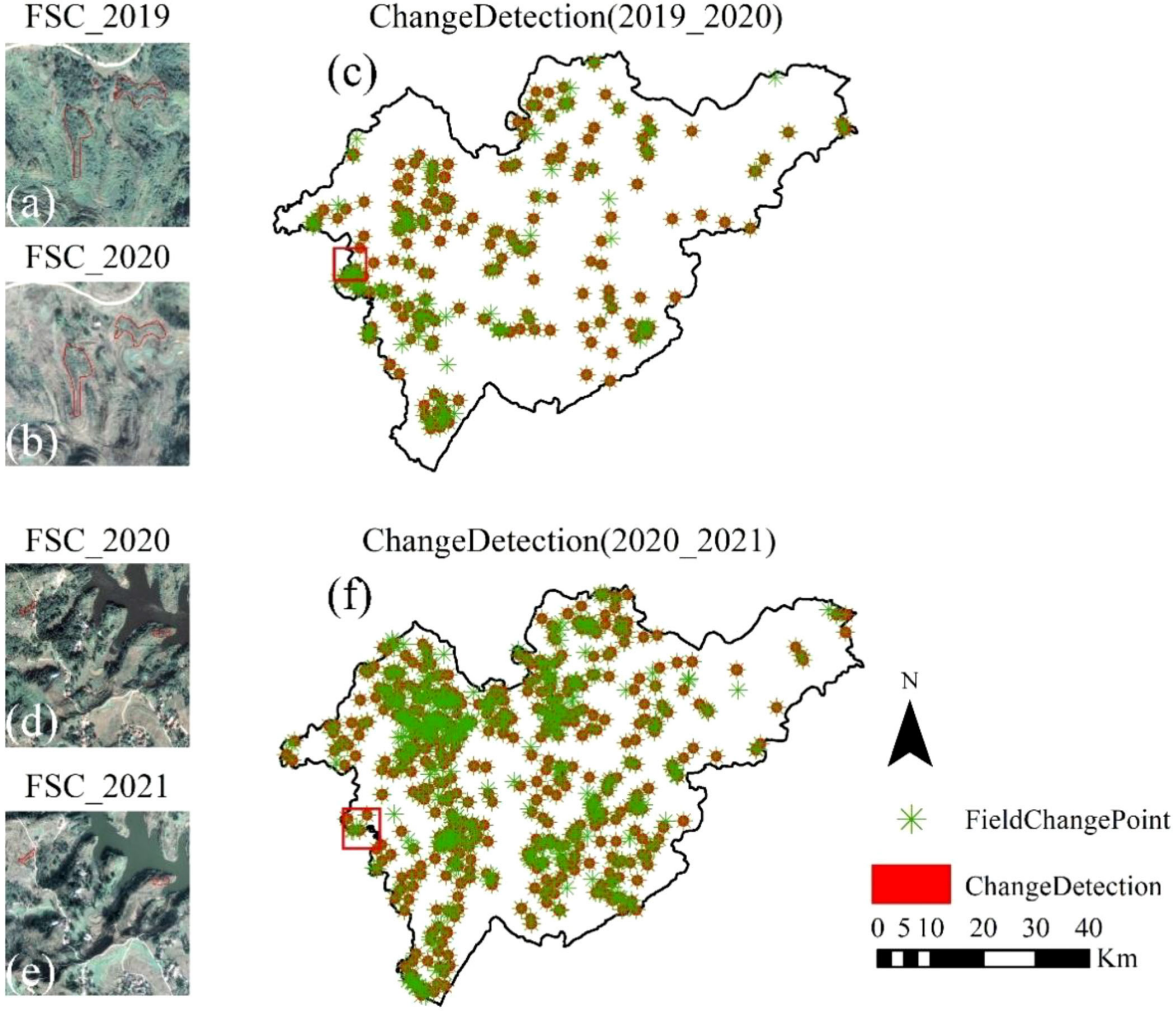
FSC-level forest cover change detection results for 2019–2020 and 2020–2021. Panels **(a, b, d, e)** show zoomed-in views of representative FSCs with detected changes, while panels **(c, f)** present the spatial distribution of FSC-level changes in the validation dataset. Green asterisks indicate field-verified change points, and red polygons denote FSC change areas identified by the change detection algorithm. Red boxes indicate selected areas containing FSC-level changes, highlighted for visual comparison.

**Figure 8** illustrates the spatial distribution and zoomed-in views of representative FSC change areas within the validation dataset between 2019 and 2021. In both periods, the detected change locations exhibit strong spatial correspondence with field survey results. During the 2019–2020 period, field surveys identified 329 FSC changes, of which 273 were successfully detected by the algorithm, while 56 were missed. Among the missed FSCs, 23 were smaller than 100 m². In the 2020–2021 period, 926 FSC changes were confirmed by field surveys, with 734 detected and 192 missed by the algorithm; notably, 130 of the missed changes were smaller than 100 m². These findings highlight the ongoing challenge of detecting small-area changes, likely due to their limited spectral and spatial signatures in remote sensing data. The detection accuracy, evaluated using OA, reached 82.7% for 2019–2020 and 79.2% for 2020–2021, satisfying the required accuracy thresholds.

Spatially, FSC changes during both 2019–2020 and 2020–2021 were mainly distributed in the western and central parts of the study area, with fewer changes observed in the eastern region. Average nearest neighbor analysis indicates that FSC changes during 2019–2020 exhibit significant spatial clustering, with a Nearest Neighbor Index (NNI) of 0.49 (p< 0.001). During 2020–2021, the number of detected FSC changes increased, accompanied by a further decrease in NNI to 0.35 (p< 0.001), indicating stronger spatial clustering. These results quantitatively describe differences in the spatial organization of FSC changes between the two periods.

### 3.4 Mapping FSC change across the entire study area

**Figure 9:**
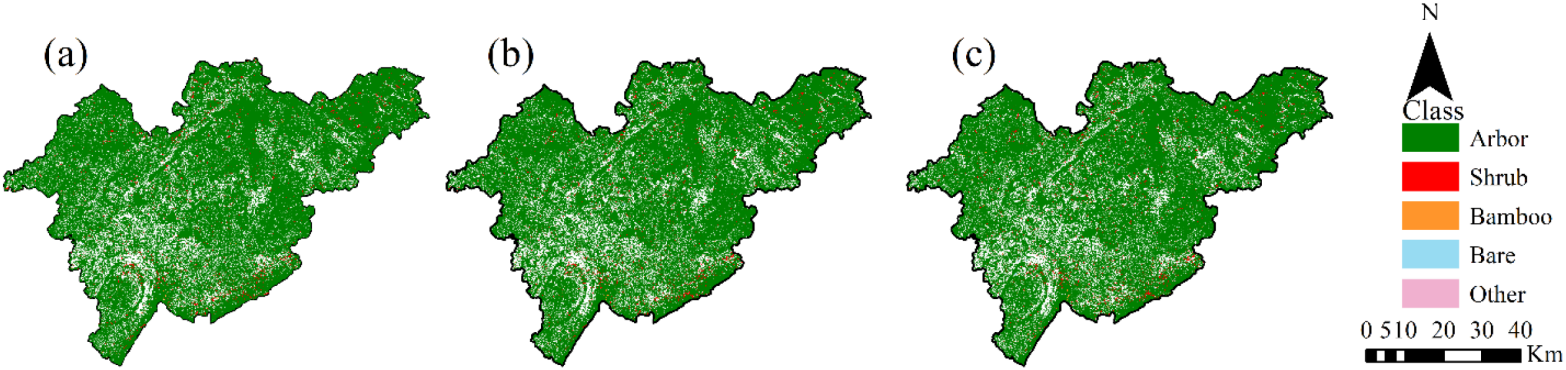
Spatial distribution of vegetation cover types in FSCs from 2019 to 2021. **(a)** 2019; **(b)** 2020; **(c)** 2021.

Additionally, vegetation cover type change results were generated and mapped for all 340588 FSCs within the entire study area. From the distribution map of FSC vegetation cover types from 2019 to 2021 (**Figure 9**), it can be seen that the spatial pattern of the main vegetation types in the study area remained stable overall, but obvious changes occurred in local areas. Complementing this, **Table 5** summarizes the number of vegetation cover type conversions among FSCs across the study area between 2019–2020 and 2020–2021, providing detailed quantitative insights into inter-type transitions over time.

**Table 5:** Number of vegetation cover type conversions in FSCs across the county from 2019–2020 to 2020–2021.

2019-2020	Arbor	Shrub	Bamboo	Bare	Other
Arbor	318154	248	231	243	231
Shrub	26	17029	25	27	25
Bamboo	5	8	2705	4	4
Bare	3	2	0	1186	3
Other	5	1	3	2	418
2020-2021	Arbor	Shrub	Bamboo	Bare	Other
Arbor	316883	352	333	311	314
Shrub	262	16945	32	28	21
Bamboo	233	33	2687	4	7
Bare	246	30	5	1176	5
Other	230	31	4	3	413

The quantitative analysis of FSC vegetation cover type transitions indicates that, overall, FSC vegetation cover types within the study area remained relatively stable across the two periods, although the degree of inter-type conversion increased in 2020–2021 compared to 2019–2020. Arbor forests demonstrated the highest persistence rates in both intervals, with a retention rate of 99.7% in 2019–2020, slightly decreasing to 99.5% in 2020–2021. Concurrently, the proportions of arbor forest converting to shrubs, bamboo, and bare land rose from 0.08%, 0.07%, and 0.08% to 0.11%, 0.10%, and 0.10%, respectively, indicating increased dynamics in arbor forest types during the latter period. Shrub retention rates decreased from 99.4% in 2019–2020 to 99.1% in 2020–2021, with the proportion of shrubs converting to arbor forest increasing from 0.15% to 0.20%. Bamboo exhibited the most pronounced changes between the two periods; its retention rate declined from 99.23% to 98.3%, while the proportion of bamboo converting to coniferous forest increased sharply from 0.18% to 8.53%, reflecting substantial vegetation type replacement in some bamboo plots during the second period. The bare land retention rate also decreased from 99.33% to 98.6%, with shrub-to-coniferous forest conversion rising from 0.25% to 0.39%, indicating an accelerated conversion of bare land to forest. Other woodland showed minor changes in retention rates, though the conversion ratios to tree and shrub types increased slightly.

In summary, vegetation cover types within the study area remained generally stable during the 2019–2020 period, with conversion rates typically below 0.3%. The primary transitions occurred among coniferous forests, shrubs, and bamboo. During 2020–2021, overall conversion rates increased, with a notable rise in transitions from bamboo to coniferous forests. According to the 2019–2021 “One Map of Forest Land” dataset, approximately 90% of the identified FSC changes are attributed to afforestation activities and tree removal associated with construction projects, while the remaining changes are mainly related to forest management practices and natural vegetation succession. These independent records provide supporting evidence for the vegetation changes observed during this period, based on documented FSC-level change reason information.

## 4 Discussion

### 4.1 Feasibility and accuracy of FSC-based forest cover change detection

Forest change detection has been widely investigated at regional to global scales, where pixel-based or grid-based approaches are commonly used to quantify overall forest dynamics (Ye et al., 2023). Such approaches are effective for large-area monitoring but are often sensitive to mixed pixels and boundary uncertainty, particularly in heterogeneous forest landscapes. In contrast, forest management and inventory systems are organized around FSCs, which serve as stable operational units (Kangas et al., 2004). Recent studies have suggested that aggregating remote sensing information at the FSC or stand level provides a more reliable analytical framework for forest monitoring, as it improves consistency with inventory data and reduces uncertainty caused by within-unit heterogeneity (Guan et al., 2023; Lv et al., 2023; Zhang et al., 2025; Wang et al., 2024).

The results of this study further confirm the feasibility of FSC-based forest cover type classification and change detection using Sentinel-2 imagery. By integrating spectral, vegetation index, and texture features within FSC units and applying a PSO- BPNN model, high classification accuracy (OA = 91%, Kappa = 0.86) was achieved, along with reliable change-detection performance at the management-unit level. The obtained performance is consistent with findings from recent fine-scale forest monitoring studies based on Sentinel-2 imagery (Alonso et al., 2023; Hu et al., 2024), indicating that FSC-based aggregation can maintain competitive accuracy while substantially improving the operational relevance of the outputs.

From an application perspective, FSC-based change detection offers clear advantages over pixel-based approaches. Because FSCs correspond directly to forest inventory and management units, detected changes can be readily interpreted and incorporated into forest resource databases, supporting tasks such as inventory updating, disturbance screening, and management decision-making. This characteristic is particularly important for large regions where field surveys are costly and where rapid identification of potential changes is required to guide further investigation (Coops et al., 2023).

### 4.2 Limitations and prospects

While the proposed FSC change detection framework demonstrates high accuracy and robustness, several limitations should be acknowledged. First, potential bias in the training dataset may influence model performance for small FSCs. In this study, FSCs smaller than 3,000 m² were excluded from model training to reduce noise and instability caused by mixed pixels. As a consequence, the trained model may exhibit reduced sensitivity to small FSCs in the validation and change detection stages. This training strategy likely contributes to the relatively high omission error observed for small-scale forest changes, particularly for FSCs smaller than 100 m², where spectral and textural signals are weak and easily masked by surrounding land cover. This limitation reflects both sample distribution bias and the intrinsic spatial resolution constraints of Sentinel-2 imagery. Second, although the PSO-BPNN model performs consistently across different vegetation types, its generalization capability in regions characterized by extremely complex terrain or highly heterogeneous forest structures remains to be further evaluated. Topographic effects, shadowing, and within-FSC variability may introduce additional uncertainty that is not fully captured by the current feature set. Third, this study relies primarily on optical remote sensing data, which are sensitive to atmospheric conditions, phenological variability, and cloud contamination. The exclusive use of optical data limits the ability to characterize forest structural changes and to detect changes under persistent cloud cover.

Future research can address these limitations in several specific ways. First, incorporating multi-source remote sensing data could improve change detection performance, particularly for small or structurally complex FSCs. For example, synthetic aperture radar (SAR) data can provide complementary information on forest structure and moisture conditions and are less affected by cloud cover, while lidar data can directly capture vertical forest structure, potentially enhancing the detection of subtle or partial canopy changes. Second, extending the analysis to longer Sentinel-2 time series beyond the 2019–2021 period would facilitate the modeling of gradual vegetation changes and reduce the influence of short-term noise and phenological variability. Time-series-based approaches may improve the discrimination between true forest cover change and temporary spectral fluctuations. Finally, future work could explore adaptive training strategies or multi-scale sampling schemes that explicitly include small FSCs, thereby improving model sensitivity to small-area changes while maintaining robustness at the management-unit level.

## 5 Conclusions

This study proposes an FSC-scale vegetation cover change detection framework that integrates ground-based survey data with Sentinel-2 imagery, providing an operational solution for forest monitoring at the management-unit level. By combining spectral bands, VIs, and texture features, an optimized multi-feature dataset was constructed, enabling the PSO-BPNN model to exploit complementary spectral, structural, and spatial information. The proposed method achieved an overall accuracy of 91% and a Kappa coefficient of 0.86, detecting approximately 80% of changed areas on an independent validation dataset.

These findings indicate that FSC-level vegetation cover changes can be efficiently identified using freely available medium-resolution satellite data, supporting prioritized field verification, timely database updates, and more efficient large-scale forest management. By focusing monitoring efforts on high-probability change areas, the method has the potential to reduce field survey costs and improve decision-making efficiency in forest management and conservation practices. However, subtle or gradual changes in FSCs are not always fully captured, which may affect transferability to dense or heterogeneous forest regions. Future work could improve detection performance and generalization by integrating additional data sources and refining feature selection and modeling strategies. Overall, this study advances a management-oriented, FSC-scale framework that complements conventional pixel-based forest change detection approaches.

## Data Availability

The datasets presented in this article are not readily available because Original FSC inventory and management data cannot be publicly shared due to institutional and governmental restrictions, and the authors do not have permission to share these data. Requests to access the datasets should be directed to WZ, zwenjie@bjfu.edu.cn.
